# The significance of region-specific habitat models as revealed by habitat shifts of grey-faced buzzard in response to different agricultural schedules

**DOI:** 10.1038/s41598-021-02315-x

**Published:** 2021-11-24

**Authors:** Kensuke Kito, Go Fujita, Fumitaka Iseki, Tadashi Miyashita

**Affiliations:** 1 Department of Ecosystem Studies, Graduate School of Agricultural and Life Sciences, The University of Tokyo, Tokyo, Japan; 2 Working Group on Threatened Wildlife, Oita, Japan

**Keywords:** Agroecology, Conservation biology, Ecosystem ecology, Macroecology

## Abstract

To determine large scales habitat suitability for focal species, habitat models derived from one region are often extrapolated to others. However, extrapolation can be inappropriate due to regional variation of habitat selection. Accounting for the ecological mechanisms causing such variation is necessary to resolve this problem. We focused on grey-faced buzzards in agricultural landscapes of Japan, which show geographically different habitat selection. To determine whether this variation is caused by the difference in climatic conditions at geographical scales or the difference in agricultural practices at smaller regional scales, we surveyed distributions of buzzards and their major prey (frogs/orthopterans) in regions differing in rice-transplanting schedules within the same climatic zone. We found that buzzards preferred paddy-forest landscapes in the early transplanting regions, but grassland-forest landscapes in the late transplanting regions. Frogs were more abundant in the early transplanting regions due to flooded paddies, while the abundance of orthopterans did not differ. The regional variation in habitat selection of buzzards may be due to different prey availabilities caused by different agricultural schedules. We propose that habitat suitability assessments of organisms inhabiting agricultural landscapes should consider differences in production systems at regional scales and such regional partitioning is effective for accurate assessments.

## Introduction

Habitat suitability models that predict the distributions of organisms based on landscape and climatic variables are useful tools for identifying areas of conservation priority^1,2^. By extrapolating the models derived from one region to other regions where existing data and research funding are insufficient, habitat suitability models are able to identify areas of conservation priority over large scales^3^. However, because habitat selection often varies spatially (mammals^4,5^; birds^6,7^; amphibians^8^), model extrapolation often fails to evaluate habitat suitability correctly^6,9^. To resolve this problem, the evaluation of habitat suitability for smaller areas (ranges) across the larger scale has been proposed^9,10^. Although previous research has partitioned these smaller ranges by latitude and longitude, and climate conditions (e.g.,^9–12^), this partitioning may not lead to correct predictions because it was not based on the ecological mechanisms that cause the spatial variation in habitat selection^9^, which are not clear in many cases.

**Figure 1:**
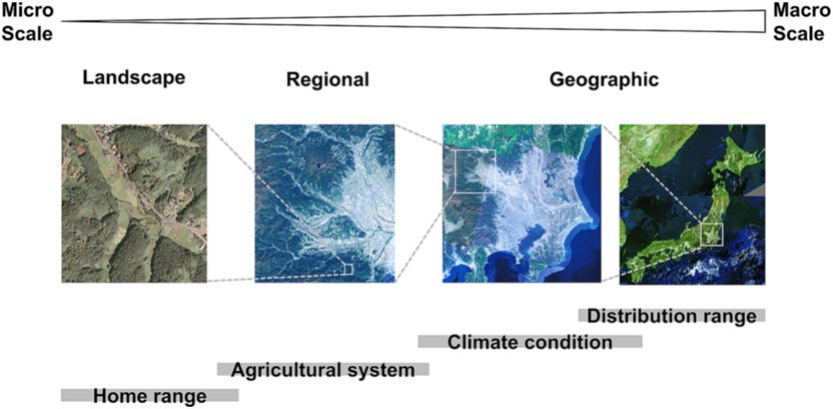
Multi-scale hierarchical structure of agricultural landscapes. The aerial photographs in the figure have been created by processing the latest seamless aerial photographs of Japan obtained from the GSI map produced by Geospatial Information Authority of Japan (https://maps.gsi.go.jp/).

It is noteworthy that the spatial variations in habitat selection can occur even between nearby regions if the key environmental conditions for focal organisms are different^3^. Especially in agricultural landscapes that are strongly influenced by anthropogenic disturbances, habitat selection is likely to differ even between close regions, because environmental conditions of agricultural landscapes vary not only by ecological factors determined at geographic scales (such as climate), but also by agricultural production systems (crop varieties, timing and cycle of production, etc.) determined at the regional socio-economic scale shown in Fig. 1^13^. Here, we use "geographic variation" to refer to the variation between regions that have different species pools in different climatic zones, and "regional variation" to refer to the variation between areas with the same species pool in the same climatic zone that occur at smaller spatial scales. In this study, we test the hypothesis that the regional variation in agricultural production systems at socio-economic scale causes different habitat selection, even in the same climatic zones.

We focused on the grey-faced buzzard (*Butastur indicus*) (hereafter buzzard), an apex predator that, in Japan, occurs primarily in agricultural landscapes. The buzzard is listed as “Vulnerable” on the Japanese Red List^14^ and has been declining in recent years due to the intensification of agricultural landscapes^15,16^. Most studies on the buzzard have been conducted in the Kanto region of eastern Japan. These studies have shown that breeding buzzards prefer mixed paddy-forest landscapes often perching on forest margins to search for and feed on frogs in paddies^17–21^. However, a recent study conducted in Fukuoka, in the Kyushu region of southern Japan, has shown that buzzards feed mainly on grassland orthopterans and prefer to breed in grassland-forest landscapes, indicating different habitat selection at the geographical scale^21^.

There are currently two proposed mechanisms that explain why such spatial variation in habitat selection for the buzzard occurs in Japan; (a) higher grassland orthopteran densities in Kyushu due to its warmer climate and (b) lower frog densities in paddy fields in the Kyushu region due to its later rice-transplanting schedule^21^. Between the two regions (Kanto vs Kyushu), climatic conditions vary at the geographic scale, while transplanting schedules vary at the regional scale, mainly due to rice marketing strategies and agricultural policies^22,23^. These two confounding effects make it challenging to determine the mechanisms by the simple comparison of habitat selection between the two regions. Here, therefore, we have chosen four study regions in the Kyushu region of southern Japan which are spatially close and in the same climatic zone, but have contrasting rice-transplanting schedules, and examined habitat selection of buzzards and their potential prey availability (frogs and orthopterans). Our general hypothesis is that the regional variation in habitat selection of buzzards is caused by different prey availabilities due to different agricultural schedules. More specific hypotheses are (a) Buzzards prefer paddy-forest landscapes in regions with early rice-transplanting schedules, while grassland-forest landscapes in regions with the late rice-transplanting schedules. (b) Densities of frogs are higher in regions with early rice-transplanting schedules than in regions with late rice-transplanting schedules, due to their preference to flooded paddy fields. (c) Orthopterans are abundant in grasslands regardless of the rice-transplanting schedules in the regions.

## Methods

### Study regions

**Figure 2:**
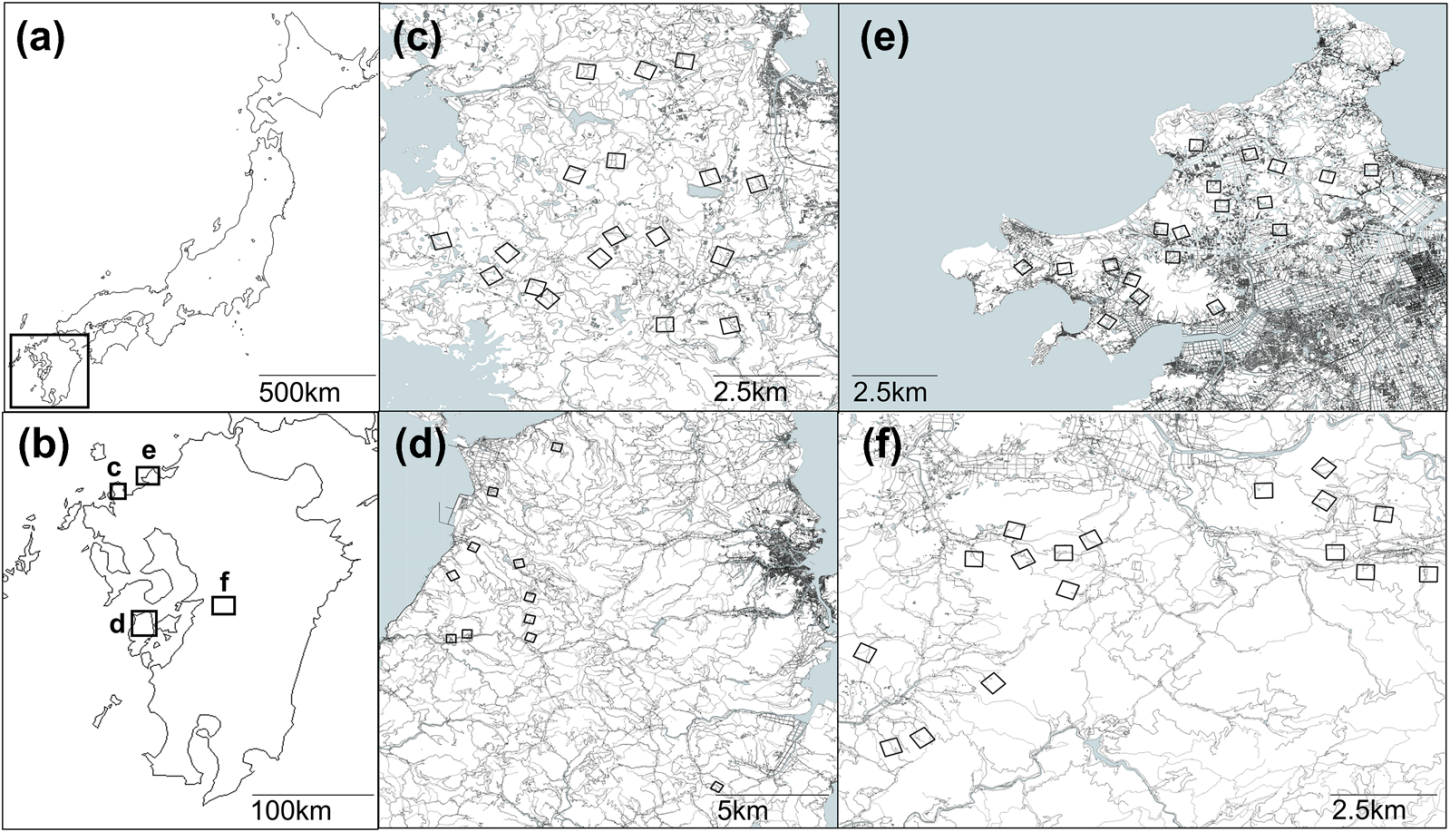
Map of (**a**) Japan and (**b**) the entire study regions, (**c**) Karatsu, (**d**) Amakusa, (**e**) Itoshima and (**f**) Uki. Squares represent study blocks. Light and dark grey areas indicate waterbodies and residential areas, respectively. Background map source is the black map of Japan (http://www.craftmap.box-i.net/japan/line.php) and the topographic data of Fundamental Geospatial Data developed by the Geospatial Information Authority of Japan (https://www.gsi.go.jp/kiban/).

We conducted the field survey in the Kyushu of southern Japan (Fig. 2a,b). The rice-transplanting schedule in Kyushu, is generally late (in late June^24^) because it is necessary to delay rice-transplanting timing after the harvest of wheat as a back crop^22,23^. However, since the technique of early transplanting was established around 1960, early transplanting (in early April^24^) has been practiced instead of a back crop of wheat in some areas in Kyushu^25,26^, so the rice-transplanting schedules differ at the regional scale. We established two study regions with early-transplanting schedules (Karatsu in northern Kyushu; N33.4, E129.9 and Amakusa in central Kyushu; N32.5, E130.1), and two study regions with late-transplanting schedules (Itoshima in northern Kyushu; N33.6, E130.2 and Uki in central Kyushu; N32.6, E130.8) (Fig. 2c–f). Each study region is 10–20 km^2^, and the distances between the regions are 20–140 km. Climatic conditions between the regions are similar (Supplementary Table S3). The size of each region is much larger than the size of the typical territory of breeding buzzards (approximately 500 m radius from the nest^18^).

### Buzzards survey

We examined the distribution of buzzards in 2019. Buzzards are migratory birds that breed in Japan, northeastern China, and the Russian Far East in summer, and overwinter in the Ryukyu Islands, Southeast Asia, and southern China^27^. In our study regions, buzzards start breeding in April, soon after returning from their wintering area. Buzzards incubate their eggs from late April until late May when they hatch. Once the eggs hatch, buzzards feed nestlings. Then nestlings start to fledge in late June, but the adults continue feeding their fledglings for several weeks. Buzzards migrate to their wintering grounds around October. The breeding season of buzzards thus overlaps largely with the rice production season, but there is a slight but significant differences in seasonality, i.e., paddies are already planted and flooded before hatching in early rice-transplanting schedules, while not yet flooded in late rice-transplanting schedules.

Because breeding buzzards were thought to prefer mosaic landscapes of farmland and forest^17,19^, we established 62 study blocks that included edges between farmland and forest in each study region (Fig. 2c–f; northern-early: 17 central-early: 11 northern-late: 17 central-late: 17). The study blocks were 400 m square and located at least 700 m apart from each other, a distance determined from the knowledge that buzzards intensively use an area of 200 m from their nests^18^. To examine the presence/absence of breeding buzzards in each block, we conducted 2 days of 30-min observations during the breeding season (April to July). Using a pilot study, we determined that this observation time was enough to minimize the possibility of missing buzzards. We identified breeding individuals based on displays, feeding behaviors, and territorial behaviors.

### Land use survey

During the brood-rearing period (late June to early July) in 2019, we recorded the land use (forest, grassland, flooded paddies, non-flooded paddies) in each block. We then used these data to create a land use map in each block using QGIS3.16.2^28^ and overlaying it on Google Earth aerial photographs in 2017.

### Prey species survey

We surveyed the distribution of prey species in paddies and grasslands in the study regions in 2019 and 2020. Based on the previous studies on the feeding habits of buzzards^17–21^, we surveyed the distribution of frogs and orthopterans larger than 3 cm as prey of this size is considered their main prey. We established survey transects in paddies and grasslands in our study blocks. We conducted surveys twice each year during the brood-rearing period (late June to early July), when breeding buzzards need a large amount of prey. We walked along the transects and counted the number of prey species observed within 0.5-m of both sides. This survey method is suitable to assess prey availability^29^ because buzzards visually search for prey (e.g.^20,30^). A total of 148 20 m-transects were placed in paddies in 34 blocks and 157 15-m transects were placed in grasslands in 37 blocks, and each transect was surveyed in both or either 2019 and 2020. In the paddy transects, we recorded the height and coverage of vegetation, the ditch characteristics (none, concrete ditch, earthen ditch), the surrounding land use (10 m width from the transect: flooded paddy, non-flooded paddy, grassland, forest, stone wall, and road), and flooding or non-flooding in the paddy field adjacent to transects. In grassland transects, we recorded the height and coverage of vegetation and the grassland types on which the transect was located (abandoned land, orchard, farmland, bank, forest edge).

### Statistical analysis

#### Buzzard model

To investigate the habitat selection of buzzards, we used a generalized linear model with a binomial error distribution. We used the edge length between the landscape elements and forest as independent variables, because the edge length, rather than the area of the landscape elements, is known to be an important determinant for buzzard distribution^19^. We prepared a land use map and calculated the edge length between the landscape elements and forest by using the field calculator of QGIS, and values were standardized (mean = 0 and SD = 1).

To explore the variation in habitat selection across regions with different transplanting schedules, we first used a model that included the interaction term of transplanting schedules and landscape elements as independent variables. The length of paddy-forest edges, grassland-forest edges, the transplanting schedules, the interaction term of the edge length and the transplanting schedules, and the study regions were included as independent variables (details of independent variables: Supplementary Table S4). The presence/absence of breeding buzzards was included as a dependent variable. We analyzed the full model and all sub-models containing different combinations of all independent variables, including the null model. We regarded models that had ΔAIC values (the difference between the AIC value of the focal model and that of the best-fit model) of < 2 as competing models and conducted model averaging^31^ for them. Model averaging was performed using the natural average method to avoid shrinkage of coefficient values^32^. Independent variables were considered influential when the 95% confidential intervals did not cross zero^32^.

As the interaction term turned out to be significant, we then analyzed the models separately for the early and the late transplanting regions. We included the length of paddy-forest edge, length of grassland-forest edge, and study region as independent variables (Supplementary Table S4), and conducted model averaging as in the first model. We confirmed that correlation coefficients of all pairs of independent variables were < 0.6, indicating no serious multicollinearity.

#### Prey species model

To clarify the relationship between frogs abundance in paddies and surrounding landscape, we used a generalized linear mixed model with a zero-inflated Poisson error distribution. We included the height and coverage of vegetation, shape of ditch adjacent to transects, surrounding land use, area of the fields with transects, flooding conditions in the fields, survey year and study region as independent variables, and study blocks as a random variables (Supplementary Table S4). We standardized the independent variables (mean = 0, SD = 1) to allow direct comparisons between estimated model effects. Since rice frogs (*Fejervarya kawamurai*) were abundant in the prey species surveys, we used the abundance of rice frogs as the dependent variable. We conducted model selection and model averaging using the same method as in the buzzard model above. We excluded models that included independent variables that have high correlation coefficients (> 0.6) to avoid serious multicollinearity.

For grasshoppers, we also used a generalized linear mixed model with zero-inflated Poisson distribution. We included the height and coverage of vegetation, land use of the grassland with transects, the survey year and the study region as independent variables, and study blocks as a random variable (Supplementary Table S4). We standardized the independent variables (mean = 0, SD = 1). Since *Gampsocleis buergeri* was by far the most abundant in the prey species surveys in grasslands, we used their abundance as the dependent variable. We conducted model selection and model averaging using the same method as the buzzards’ models. As above, we excluded the models that included independent variables that were highly correlated (> 0.6) to avoid serious multicollinearity.

We performed all analyses in R 4.0.3^33^, using the glmmTMB packages^34^ for model fitting, the MuMIn package^35^ for model selection and averaging, and the ggplot2^36^ for graphic illustration or results.

## Results

### Buzzards

**Figure 3:**
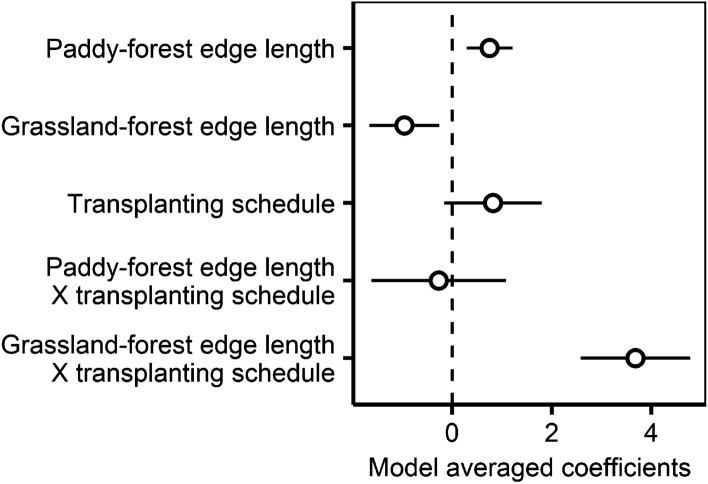
The estimated model averaged coefficients of the buzzards model that included interaction term. Error bars represent standard errors.

**Figure 4:**
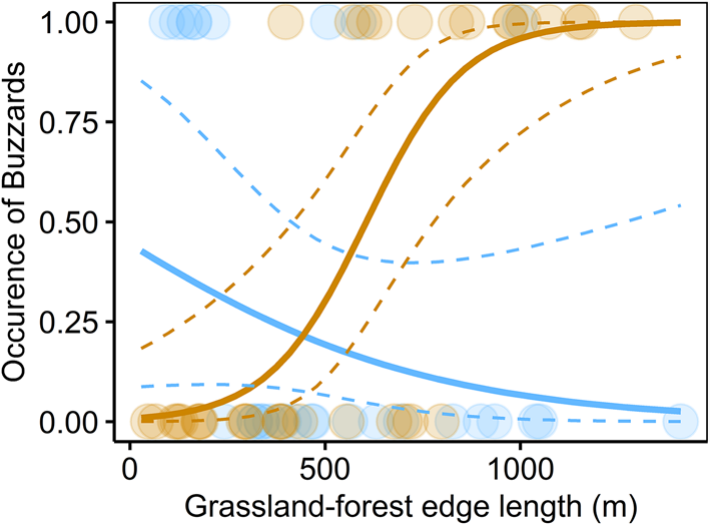
The relationship between breeding buzzards and grassland-forest edge length. Blue dots represent the data in early transplanting regions and yellow dots represent the data in late transplanting regions. The solid lines represent the regression curve estimated from the smallest AIC model including interaction term and the dotted lines represent the 95% credible interval.

Breeding buzzards were found in 23 of the 62 blocks (northern-early region; 5, central-early region; 5, northern-late region; 8, central-late region; 5). The probability of the presence of buzzards in each block varied regionally (northern-early region: 0.29, central-early region: 0.45, northern-late region: 0.47, central-late region: 0.29) but did not differ depending on the regional transplanting schedules. For the model analysis that included the interaction term, ΔAIC of the null model was 24.3, and there were three competing models with ΔAIC of < 2. The top model included four independent variables: paddy-forest edge length, grassland-forest edge length, transplanting schedules, and the interaction term of grassland-forest edge length and the transplanting schedules (Supplementary Table S1). The model averaging of the competing models (Supplementary Table S1, Fig. 3) showed that the interaction term between grassland-forest edge length and transplanting schedules had a positive effect (Estimate ± SD: 3.68 ± 1.10). This implies that the relationship between buzzards and grassland-forest edge length is different between the early transplanting and late transplanting regions (Fig. 4).

**Figure 5:**
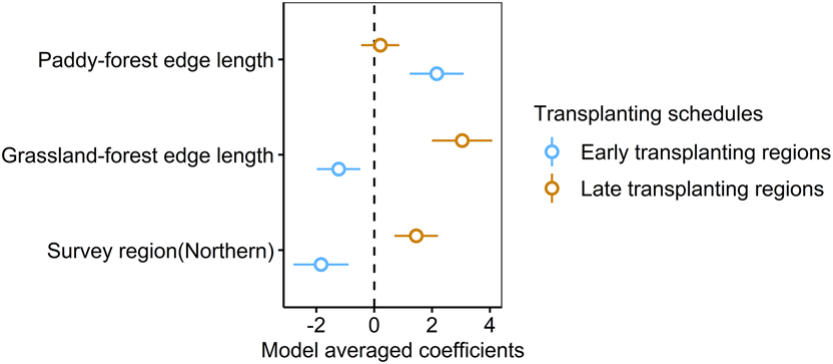
The estimated model averaged coefficients of the buzzards model in the early and late transplanting regions. Error bars represent standard errors.

**Figure 6:**
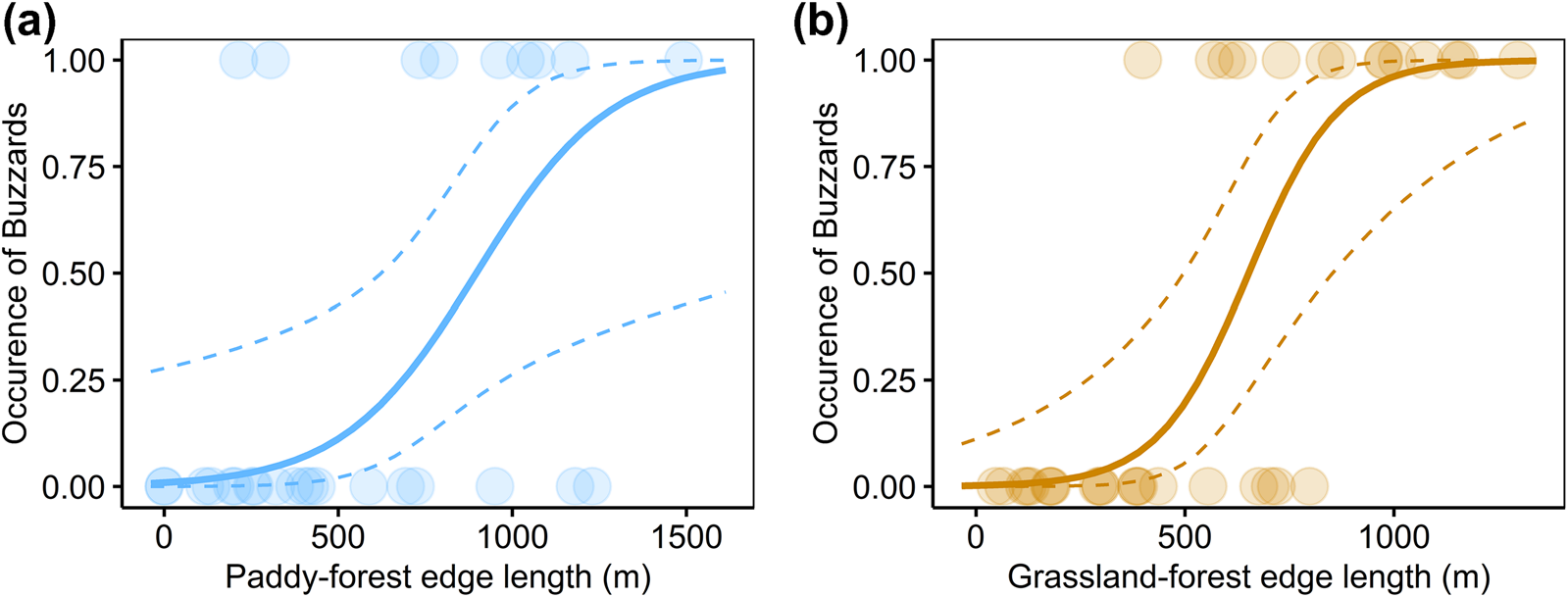
(**a**)The relationship between breeding buzzards and paddy-forest edge length in early transplanting regions, and (**b**) the relationship between breeding buzzards and grassland-forest edge length in late transplanting regions. Dots represent the data of each blocks. The solid lines represent the regression curve estimated from the smallest AIC model in the early and late transplanting regions and the dotted lines represent the 95% credible interval.

We next developed models separately for early and late transplanting regions. For the early transplanting regions (Supplementary Table S2), the ΔAIC of the null model was 9.0, and there were two competing top-ranked models. The top model included paddy-forest edge length, grassland-forest edge length, and survey region as independent variables (Supplementary Table S2). The model averaging showed that the paddy-forest edge length had a positive effect (Estimate ± SD: 2.16 ± 0.93; Figs. 5, 6a). For the late transplanting regions (Supplementary Table S2), the ΔAIC of the null model was 24.7, and there were two competing models. The top model included grassland-forest edge length, and survey region as independent variables (Supplementary Table S2). The model averaging showed that the grassland-forest edge length had a positive effect (Estimate ± SD: 3.04 ± 1.04; Figs. 5, 6b).

### Prey species

**Figure 7:**
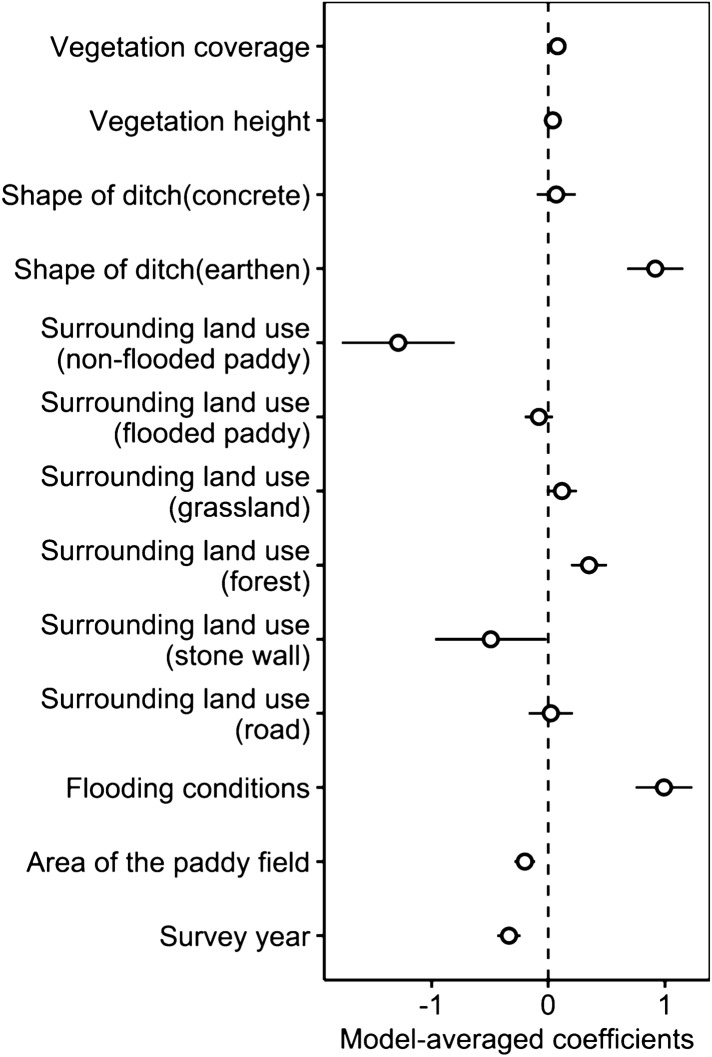
The estimated model averaged coefficients of the prey species model in paddies. Error bars represent standard errors.

The prey survey in the paddies showed that the rice frog (*F. kawamurai*) was by far the most abundant (mean abundance in each transect: 2.67 ± 3.02 individuals) in comparison to the black spotted pond frogs (*Rana nigromaculata*; 0.08 ± 0.31), the Japanese tree frogs (*Dryophytes japonica*; 0.01 ± 0.12), and the Japanese red frogs (*Rana japonica*; 0.01 ± 0.07). The abundance of rice frogs was higher in the early transplanting regions (northern region: 3.28 ± 3.52 individuals, central region: 3.28 ± 2.64 individuals) than in the late transplanting regions (northern region: 1.59 ± 2.29 individuals, central region: 2.74 ± 3.14 individuals). For the analysis of the relationship between rice frogs and land use, ∆AIC of the null model was 49.3, and there were 10 competing models. The model averaging (Fig. 7) showed that flooding in the fields and earthen ditches had positive effect, while surrounding dry (non-flooded) paddies and forest, and the area of paddy field had negative effect. Also, earthen ditches had positive effect, and the size of the paddy field and forest as surrounding land use had negative effect.

**Figure 8:**
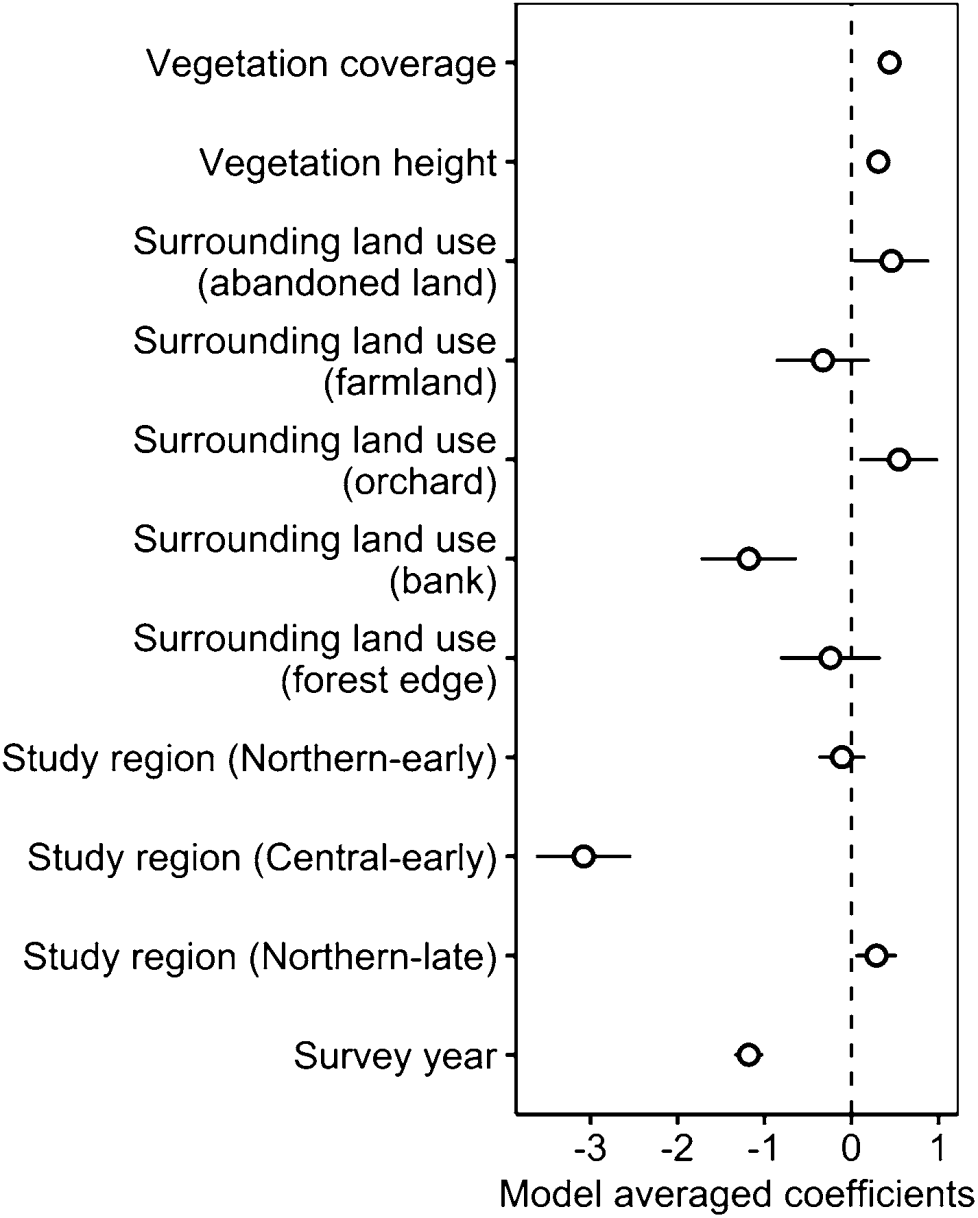
The estimated mode averaged coefficients of the prey species model in grasslands. Error bars represent standard errors.

The prey survey in the grasslands showed that *G.buergeri* was by far the most abundant (mean number in each transect: 2.00 ± 3.24 individuals), while *Tettigonia orientalis* (0.01 ± 0.14), *Locusta migratoria* (0.03 ± 0.18), and *Euconocephalus thunbergi* (0.00 ± 0.14) were much less abundant. For the model analysis of the relationship between *G. buergeri* and land use, the ∆AIC of the null model was 113.3, and there were 13 competing models. The model averaging (Fig. 8) showed that the central-early region had an negative effect, but the abundance did not depend on the regional transplanting schedules. Also, vegetation cover and height had a positive effect, and abandoned land and orchards had a positive effect.

## Discussion

### Ecological correlates with regional variation in buzzard habitat selection

Our study showed that the habitat selection of buzzards depended on the regional transplanting schedules of rice paddies. Within the same climatic zone, buzzards preferred paddy-forest landscapes in early transplanting regions and grassland-forest landscapes in late transplanting regions. This difference may be due to the contrasting prey availability in the contrasting landscapes. The abundance of frogs was higher in early transplanting regions because frogs prefer flooded paddies, while grassland orthopteran abundance did not depend on transplanting schedules. Therefore, buzzards appear to use paddy-forest landscapes to feed on frogs in early transplanting regions, but they use grassland-forest landscapes to feed on orthopterans in late transplanting regions. Although we did not examine the feeding habits of buzzards in this study, the above inference is supported by previous studies; frogs are the main prey species in early transplanting regions in eastern Japan^17–21^ and orthopterans are the main prey species in regions with late transplanting schedules such as Itoshima (Iseki, unpublished) and in wintering areas such as Ryukyu Islands, where frogs hibernate^37^.

It should be noted, however, that paddy-forest landscapes in early transplanting regions may be more suitable as breeding habitat than grassland-forest landscapes in late transplanting regions. The reason why flooded paddies are more suitable for buzzards than grasslands is that frogs may be a more preferable prey than orthopterans if both are available. Although the density of frogs in paddies in early transplanting regions and that of orthopterans in grasslands in late transplanting regions was not markedly different, the wet weight of a 3 cm frog is estimated to be 2.4 g^38^ compared to 1 g for a same sized orthopteran^39^. Additionally, the net ecosystem production in paddy fields is higher than in grasslands due to faster carbon cycle in flooded fields with high water temperatures^40–42^. This may provide more potential prey biomass in flooded paddies than in dry grasslands. The difference in prey availability for buzzards, therefore, would be expected to result into higher reproductive success and hence local density.

However, we found no significant difference in the density of buzzards among regions with different transplanting schedules. It is well known that population density may not correlate with habitat suitability^43,44^. One reason for this is that territorial competition between individuals often limits population densities^43^. Previous studies on raptors have shown that competitively superior individuals occupy habitats with high habitat suitability, while competitively inferior individuals occupy less ideal habitat^45,46^. Competition among buzzard individuals in high quality landscapes may force some individuals to move to suboptimal landscapes, resulting in no difference in breeding density between early transplanting and late transplanting regions. More intensive studies regarding reproductive success of buzzards in different regions are required in the future to determine whether grassland habitats contain competitively inferior individuals with lower breeding success. It is important, however, to acknowledge that if grassland habitats were determined to be suboptimal, it does not mean that this landscape element has a lower conservation value. As demonstrated in the famous metapopulation model^47^, suboptimal habitats have a crucial role in increasing population size at the regional level. Grasslands in Japan have been maintained by human management such as mowing, burning and grazing^48^, but they are severely declining due to land abandonment^49^. Maintaining human management of grasslands in agricultural landscapes is thus essential to conserve buzzard populations.

### Habitat suitability assessment and conservation of buzzards

Given the finding that habitat selection differs depending on regional transplanting schedules, we propose that habitat suitability for buzzards should be evaluated in each range partitioned by the transplanting schedules at the regional scale. In contrast to previous studies which propose range partitioning by latitude and longitude and climate conditions, this is a more appropriate range partitioning that is based on the understanding of ecological mechanisms. As information on the regional transplanting schedules is readily available by the government^24^ and local agricultural organizations, this partitioning criterion could be achieved easily by scientists and decision makers.

In a more general sense, the above approach that partitions ranges by regional differences in cropping systems can be easily applied to future change of agricultural landscapes. Habitat suitability assessments that consider the impact of future climate change have been studied in recent years, but few studies have incorporated landscape changes due to human response to climate change or socio-economic change^50^. Agricultural production systems are particularly changeable, which can significantly alter agricultural landscapes. For example, changes in agricultural schedules including grassland mowing^51^ and rice-transplanting^52^ are ongoing, and crop changes that are suitable to local climate change and those used for fuel production (energy crops) are increasing^53^. As the distribution of animals inhabiting agricultural landscapes are likely to be influenced by such changes, regional partitioning based on the future land use scenarios will be a useful way to predict habitat suitability under future climate change scenarios.

The final point to note is that the effect of regional difference in agricultural schedules on habitat selection of animals may be changeable and context-dependent at larger geographical scales across different climatic zones. In eastern Japan, which is in a cool temperate zone, rather than a warm climate zone as in Kyushu, species richness of orthopterans is comparatively lower^54^, and developmental phenology is retarded^55^. Therefore, grasslands may not be suitable habitat in late transplanting regions in these cooler climates, due to the limited orthopteran availability. In fact, few breeding buzzards are recorded in Saitama, Gunma, and Kanagawa, all cooler areas where transplanting schedules are late in the eastern Japan^56^. It is necessary, therefore, to consider the differences in species pools and species phenology of prey species at the geographic or biome scale, and then to consider the effects of differences in socio-economic factors at the regional scale on food availability. Although it has long been argued that species-landscape relationships are scale dependent^57,58^, assessing habitat suitability at multiple hierarchical scales is still a challenge^59^. Our study suggests that in socio-ecological production landscapes, such as agricultural landscapes, habitat suitability assessment may need to be conducted at two hierarchical scales: the geographic scale with different ecological conditions, and the regional scale with different socio-economic conditions. We believe that such considerations will allow us to estimate suitable habitat for focal organisms at a range of spatial scales in a meaningful and robust manner. This is particularly important in the face of a changing world.

## Supplementary Information

Supplementary Table S1.

Supplementary Table S2.

Supplementary Table S3.

Supplementary Table S4.
